# Expression Patterns of Non-Coding Spliced Transcripts from Human Endogenous Retrovirus HERV-H Elements in Colon Cancer

**DOI:** 10.1371/journal.pone.0029950

**Published:** 2012-01-06

**Authors:** Qiaoyi Liang, Zefeng Xu, Rongzhen Xu, Lijun Wu, Shu Zheng

**Affiliations:** 1 Cancer Institute, The Second Affiliated Hospital, Zhejiang University School of Medicine, Hangzhou, Zhejiang, China; 2 Department of Medicine and Therapeutics, Li Ka Shing Institute of Health Sciences, The Chinese University of Hong Kong, Hong Kong, China; 3 Key Laboratory of Cancer Prevention and Intervention, China National Ministry of Education, Key Laboratory of Molecular Biology in Medical Sciences, Zhejiang Province, China; University of Saarland Medical School, Germany

## Abstract

**Background:**

Up-regulation of the most abundant H family human endogenous retrovirus (HERV-H), especially env-related transcripts, correlates with colon cancer. However, expression pattern of spliced non-coding transcripts of HERV-H is not clear.

**Methodology/Principal Findings:**

In this study, expression of HERV-H spliced transcripts in colon cancer was investigated by a RT-PCR strategy using primers targeting the tRNA^His^ primer-binding site and the R region in the 3′ long terminal repeat (LTR), followed by cloning and sequencing of the amplicons. Sequences were then assigned to individual HERV-H loci by employing private nucleotide differences between loci. Different expression patterns of HERV-H spliced transcripts from distinct active elements were found in colon cancer cell lines HT29, LS 174T, RKO, SW480 and SW620. Furthermore, the expression patterns in SW480 and RKO were significantly changed by demethylation treatment. Interestingly, more HERV-H elements were found to be transcriptionally active in colon tumor tissues than in adjacent normal tissues (14 *vs.* 7).

**Conclusions/Significance:**

This is the first research to study the character of expression of non-coding spliced transcripts of HERV-H elements in colon cancer. Expression patterns of HERV-H spliced transcripts differed among colon cancer cell lines and could be affected by genomic DNA methylation levels. More importantly, besides the commonly accepted view of up-regulation of HERV-H expression in colon tumor tissues, we found more active HERV-H loci in colon tumor as compared with adjacent normal tissues.

## Introduction

Human endogenous retroviruses (HERVs) constitute 8% of the human genome [1]. They are inferred to originate from germ-cell infection by exogenous retroviruses during primate evolution [2]. The proviral structure of HERV elements mainly consists of 5′ LTR-*gag*-*pro*-*pol*-*env*-3′ LTR, in which the four genes (*gag*: group-specific antigen, *pro*: protease, *pol*: polymerase, and *env*: envelope) encode structural/functional proteins essential to a replication-competent retrovirus, and the long terminal repeats (LTR) at both ends differ HERVs from other retrotransposons such as long interspersed nuclear elements (LINEs). Most of the remnants of HERVs are simply isolated LTR copies, with the internal sequence having been lost during integration or via homologous recombination. HERV families are defined by different criteria, such as homogeneity to their exogenous counterparts, sequence similarity of the *pol* genes, and the primer binding site (PBS) immediately downstream of the 5′ LTR. H family HERV (HERV-H) contains a PBS with a sequence similar to human tRNA^His^. There are about one thousand HERV-H elements throughout the human genome, and most of them (800–900) lack almost the entire *env* region [3], [4]. It has been reported that only 18 HERV-H elements in the human genome are relatively complete [5], and only three were identified to contain intact *env* open reading frames (ORFs) [6].

Although most of the HERV-H elements are structurally incomplete due to mutation and deletion during evolution, there are hundreds of them retaining complete 5′ and 3′ LTRs and a PBS-tRNA^His^ immediately downstream of the 5′ LTR [7]. The expression of HERV elements is mostly under the control of their LTRs [8]. A complete LTR element has a structure of U3-R-U5 (5′→3′), containing both transcription initiating and terminating signals. The transcription of HERV elements usually starts from the R region in the 5′ LTR and terminates at the end of the R region in the 3′ LTR. It is also proposed that cell type-dependent expression of HERV elements is usually controlled by specific regulatory sequences located mainly in the U3 region [9].

Due to the immunosuppressive property of the envelope protein of HERV-H, many studies focused on the *env*-related transcripts [10], and those non-*env*-related transcripts were rarely paid attention to. Up-regulation of the most abundant H family HERV, especially *env*-related transcripts, has been reported to be associated with colorectal cancer [5], [11], [12]. In our previous study, we observed that there were many spliced non-coding RNA transcribed from HERV-H elements, both in normal and cancerous colon tissues, as well as colon cancer cell lines. However, the expression pattern of the spliced non-coding transcripts from HERV-Hs is not clear. During the studies on the three newly identified HERV-H-related genes [13], [14], [15] in our laboratory, we observed that the overall expression of HERV-H elements in colon cancer was complex and different between tumor samples and adjacent normal samples. We also observed that many *env*-deleted HERV-Hs were transcriptionally active, and many spliced non-coding RNA were transcribed in both tumor and normal tissues, as well as cancer cell lines. In this study, we initiated the first study to find out the exact loci of the most active HERV-H elements in colon cancer.

## Results

### The RT-PCR-sequencing strategy for hooking HERV-H non-coding transcripts

**Figure 1:**
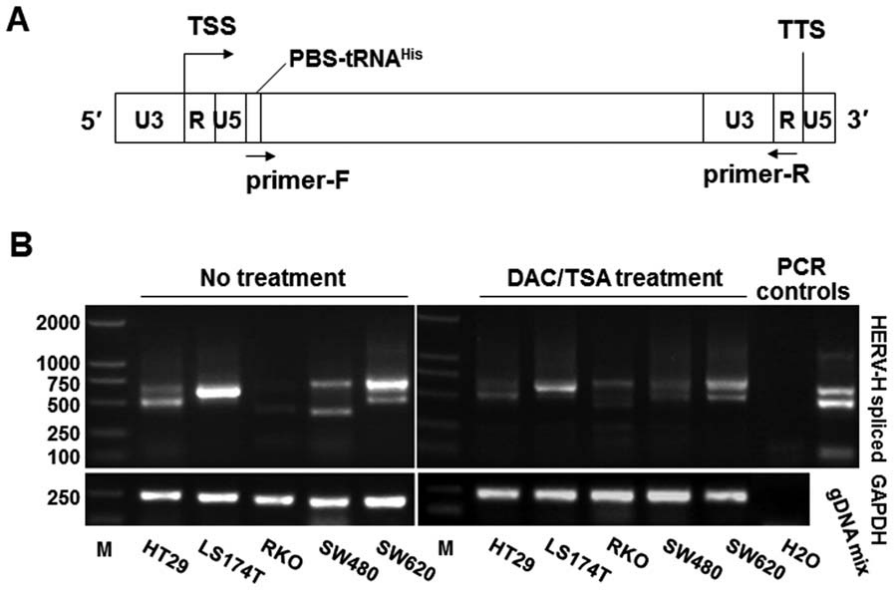
RT-PCR detection of HERV-H spliced transcripts in colon cancer cell lines. **A.** Schematic demonstration of the primer locations. TSS, transcription start site; TTS, transcription termination site. **B.** RT-PCR was performed to detect HERV-H spliced transcripts in colon cancer cell lines with/without demethylation/histone acetylation treatment using the DNA demethylation agent DAC and the histone deacetylase inhibitor TSA. H2O and genomic DNA mixture (gDNA mix) were used as controls. Genomic DNA mixture produced bands distinct from cDNA samples, which were reverse transcribed from DNase-treated RNA.

Since there are over one thousand members of the HERV-H family throughout the human genome, with sequences of low complexity and homologous to one another, it is difficult to study their transcription profile by taking every member into consideration. In order to profile the expression of HERV-H in colon cancer cell lines, colon tumor and adjacent normal tissues, and to identify the exact loci of the most active HERV-H elements, we developed a RT-PCR-cloning strategy to hook a selected group of HERV-H-related transcripts. The transcription of HERV elements is typically started from the 5′ R region and terminated at the end of the 3′ R region [16]. We designed the primers by referring to the sequence of the HERV-H consensus constructed by Jern *et al.* [5], with the forward primer targeting the PBS-tRNA^His^ downstream of the 5′ LTR and the reverse primer targeting the R region of 3′ LTR (Fig. 1A). This pair of primers was supposed to generate PCR products from HERV-H transcripts of mixed sizes. Consequently, the total RT-PCR products were cloned into the pGEM-T Easy vectors and transfected into *E. coli*. More than thirty colonies of each sample were randomly selected and plasmids were then sequenced. Sequencing results were assigned to specific HERV-H loci based on private nucleotide differences between individual loci (one or several nucleotides that are characteristic for an HERV-H locus). Sequencing results of all the randomly selected colonies indicated that no inserts were from non-HERV-H transcripts, demonstrating our strategy worked well.

### Expression of HERV-H spliced transcripts in colon cancer cell lines, and altered expression in response to DNA demethylation treatment

RT-PCR assays were performed on five colon cancer cell lines, including HT29, LS 174T, RKO, SW480 and SW620. Different band patterns were obtained from these cell lines (Fig. 1B left). A relatively lower level of HERV-H expression was observed in RKO. It has been reported that there is DNA methylation at a global genomic level by targeting repetitive sequences [17], so we further investigated whether demethylation treatment would change the transcription pattern of HERV-H elements. Cancer cells were treated with DNA methylation and histone deacetylase inhibitors (DAC and TSA respectively). RT-PCR results showed that expression patterns of HERV-H were significantly changed in SW480 and RKO cells (Fig. 1B right). These findings suggested that the epigenetic context of a genome affected the expression of some of the HERV-H elements.

RT-PCR product of each colon cancer cell line was further analyzed by sequencing after cloning. Sequencing results were assigned to genomic loci by BLAT searches against the human genome assembly (hg19). Genomic DNA sequences were then retrieved and analyzed with RepeatMasker to determine the HERV-H sequences. Only a limited number of HERV-H elements were found to be transcriptionally active in each of these cell lines, with six loci (seven elements) in HT29 and two or three in each of the other cell lines (Table 1).

**Table 1:** The loci of active HERV-H elements in colon cancer cell lines.

Cell lines	Loci of active HERV-Hs (hg19)		Size/bp	Predicted ORFs (Size/nt, homologous protein)***
HT29	1p32.3*	Chr1+: (53890009–53895701)∼(53898553–53900850)	(5693)∼(2298)	426, Gag protein
	2p14	Chr2−: 69670143–69676473	6331	540, Polymerase
	3q28	Chr3+: 189862389–189867991	5603	900, partial Gag protein
	14q24.3	Chr14+: 74170042–74175916	5875	
	16q24.1**	Chr16+: 86311701–86314885	3185	495, Integrase; 618, conserved domain RT-like
	19q13.32	Chr19−: 47553126–47558666	5541	
LS174T	22q11.1	Chr22+: 17092198–17096039	3842	825, Gag protein
	19q13.31**	Chr19−: 43827663–43833536	5874	
RKO	3q24	Chr3+: 145428282–145433882	5601	
	6q24.1	Chr6+: 142336803–142342919	6117	525, conserved domain CREB5
	19q13.41	Chr19+: 53131048–53136648	5601	
SW480	1q25.3	Chr1+: 183582345–183588508	6164	
	6q24.2	Chr6+: 145244311–145249992	5682	
	20p12.1**	Chr20−: 12320915–12326587	5673	
SW620	1q25.3	Chr1+: 183582345–183588508	6164	
	5q31.1	Chr5−: 135066688–135073175	6488	528, Gag protein; 747, conserved domain RT-like

*Two adjacent HERV-H elements at 1p32.3 were combinedly active by making use of 5′ LTR of the first HERV-H and 3′ LTR of the second one (representative transcript sequence JK017392).

**The elements located at 16q24.1 and 19q13.31 are also actively transcribed in both tumor and adjacent normal colon tissues, while the one located at 20p12.1 is active in tumor tissue.

***Open reading frames (ORFs) were predicted by the online program ORF Finder and putative peptide sequences were subjected to Blastp search against the Non-redundant protein sequences at http://www.ncbi.nlm.nih.gov/. Only predicted ORFs≥303 nt (peptide sequence ≥100 aa) and with Blastp matches are included. Gag, group-specific antigen; RT, Reverse transcriptase; CREB5, cAMP response element-binding protein 5.

### Characterization of the active HERV-H elements and their spliced transcripts in colon cancer cell lines

**Figure 2:**
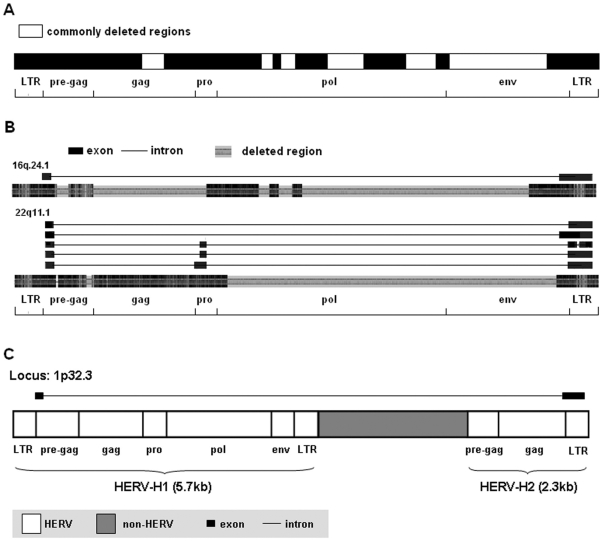
Characterization of the active HERV-H elements in colon cancer cell lines and their spliced transcripts. **A.** Schematic of the six commonly deleted regions in the active HERV-H elements in colon cancer cell lines. **B.** Schematics of the two extraordinarily short HERV-H elements and their transcripts. Pair-wise alignments for each HERV-H element were performed with the HERV-H consensus constructed by Jern P, *et al*. The shortened alignment results were shown to indicate the missing regions precisely. Color density represents the extent of homology with the HERV-H consensus. Gray areas represent deleted regions in the HERV-H elements as compared with the HERV-H consensus. Spliced transcripts are shown above the alignment results accordingly. Thick bars represent exons, and lines represent introns. Regions of LTRs, pre-gag, *gag*, *pro*, *pol* and *env* are labeled below. **C.** Schematics of the two combinedly active HERV-H elements located at 1p32.3 in HT29.

Pair-wise alignments were performed with the 9.0-kb HERV-H consensus constructed by Jern *et al.* to determine the fragment-deleting patterns of each HERV-H element. Alignment results showed all the HERV-H elements active in colon cancer cell lines were structurally incomplete, with the longest one being 6,488 bp long (Table 1 and Fig. S1). Six fragments were found to be commonly deleted in these elements, including a short one in the *gag* region, four in the *pol* region, and nearly the entire *env* region (Fig. 2A). There were some exceptions. The element located at 16q24.1 (active in HT29) and the one located at 22q11.1 (active in LS 174T) were extraordinarily short (Fig. 2B). The one at 16q24.1 was only 3,185 bp in length, with the whole *gag* region and a large fragment encompassing the *pol* and *env* deleted. The one at 22q11.1 was 3,842 bp long, with nearly the entire *pol* and *env* regions deleted. Most of the amplicons (>90%) from LS 174T contained sequences corresponding to spliced RNAs from the 3.8-kb HERV-H element at 22q11.1, most of which (>60%) were multiply spliced. Interestingly, two HERV-H elements located at 1p32.3 (2.8 kb separated) were found to be active in HT29 cells, making use of 5′ LTR of the first HERV-H and 3′ LTR of the second one (representative transcript sequence JK017392). Sequence analysis revealed that 5′ LTR was missing from the latter 2,298-bp HERV-H element (Fig. 2C).

### More HERV-H elements were transcriptionally active in colon tumor than in adjacent normal tissues

**Figure 3:**
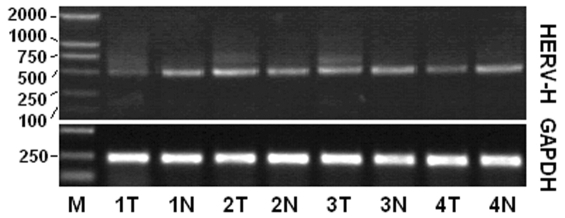
Gel electrophoresis of RT-PCR products of HERV-H-related transcripts in colon tumor and adjacent normal tissues. M, DL2000 ladder; T, tumor; N, normal.

Expression of HERV-H transcripts in colon tumor and adjacent normal tissues were analyzed by RT-PCR. A slight difference between the RT-PCR products of colon tumor and adjacent normal samples was observed, with some products of larger sizes present in tumor samples (Fig. 3). After sequencing and sequence analysis of the PCR products, 14 HERV-H elements were found to be transcriptionally active in colon tumor samples. In contrast, only 7 HERV-H elements were found to be active in the adjacent normal colon samples. Details of these HERV-H elements were indicated in Table 2. Sequence analysis by pair-wise alignment against the HERV-H consensus revealed that all the active HERV-H elements were structurally incomplete, with six fragments commonly deleted as indicated in Fig. 2A (Fig. S2).

**Table 2:** The loci of active HERV-Hs in colon tumor and adjacent normal tissues.

Colon tissues	No.	Loci of active HERV-Hs (hg19)*		Size/bp	Abundance (>10%)/*Notes*		Predicted ORFs (Size/nt, homologous protein)****
**Tumor**	T1	**1q42.2**	Chr1+: 230736962–230744940	7979	17.78%	**68.89%**	399, conserved domain RT-like; 402, Envelope
	T2	**16q24.1**	Chr16+: 86311701–86314885	3185	26.67%		618, conserved domain RT-like
	T3	**19q13.31**	Chr19−: 43827663–43833536	5874	11.11%		318, CREB5
	T4	**1p31.3**	Chr1+:68851687–68857675	5989	13.33%		
	T5	2p12	Chr2−: 76800751–76806830	6080			
	T6	3q26	Chr3+: 189862389–189867991	5603			
	T7	8q22.2	Chr8+: 100955923–100961835	5913			
	T8	11q24.2	Chr11−:127638814–127644790	5977			741, Polymerase
	T9	13q33.3	Chr13+: 109917438–109923464	6027			327, Protease
	T10	20p12.1	Chr20−: 12320915–12326587	5673			
	T11	5q23.2	Chr5−: 121809921–121814541	4621	*5′LTR incomplete*		
	T12	Xp22.32	ChrX−: 4458515–4464361	5847	*HERV-HX* **		
	T13	1q31.3	chr1−:195817044–195817727	684	*5′LTR-3′LTR* ***		
	T14	12p12.2	Chr12−:20970842–20975551	4710	*3′LTR missing*		327, conserved domain Protease; 309, conserved domain RT-like
**Adjacent Normal**	N1	**1q42.2**	Chr1+: 230736962–230744940	7979	26.19%	**85.71%**	(as T1)
	N2	**16q24.1**	Chr16+: 86311701–86314885	3185	16.67%		(as T2)
	N3	**19q13.31**	Chr19−: 43827663–43833536	5874	42.86%		(as T3)
	N4	4p15.2	Chr4−: 23724496–23729489	4994			
	N5	19q13.12	Chr19−:36750410–36756122	5713			309, conserved domain RT-like
	N6	Xq23	ChrX−: 115854285–115860680	6396			564, Gag protein
	N7	5q23.2	Chr5−: 121809921–121814541	4621			(as T11)

*Active elements individually contributing to more than 10% of the transcripts in tumor or adjacent normal samples are highlighted in boldface and their transcript abundances are indicated in the ‘Abundance’ column (individual and total, respectively).

**HERV-HX is the colon cancer-related HERV-H element identified by us previously [14]. Inserts of PCR product clones were all HERV-HX fragments but not spliced sequences, in concordance with our previous finding that no spliced transcripts were produced from HERV-HX in colon tumor samples.

***The element located at 1q31.3 consists of 5′LTR and 3′LTR, with the entire protein coding region (*gag-pro-pol-env*) missing.

****Open reading frames (ORFs) were predicted by the online program ORF Finder and putative peptide sequences were subjected to Blastp search against the Non-redundant protein sequences at http://www.ncbi.nlm.nih.gov/. Only predicted ORFs≥303 nt (peptide sequence ≥100 aa) and with Blastp matches are included. RT, Reverse transcriptase; CREB5, cAMP response element-binding protein 5; Gag, group-specific antigen.

Four HERV-H elements, located at 1q42.2, 16q24.1, 19q13.31 and 5q23.2 respectively, were active in both tumor and adjacent normal tissues. We further analyzed the transcript abundance from each active HERV-H element. Interestingly, three of the commonly active elements (located at 1q42.2, 16q24.1 and 19q13.31) each contributed to more than 10% of the transcripts in both tumor and adjacent normal samples. There was another element, located at 1p31.3, producing more than 10% of the transcripts in tumor. The four most active elements in the tumor sample, i.e. those located at 1q42.2, 16q24.1, 19q13.31 and 1p31.3, in total contributed to 68.89% of the transcripts in the tumor sample. By contrast, the three most active elements in normal samples (located at 1q42.2, 16q24.1 and 19q13.31) contributed to 85.71% of the overall transcripts. These results indicated that there were more HERV-H elements active in colon tumor tissues than in adjacent normal tissues. Furthermore, the majority of transcripts in adjacent normal samples were produced from fewer HERV-H elements when compared with tumor samples.

## Discussion

Due to the abundance of HERV-H elements in the human genome and their high sequence similarity to each other, there seems to be no appropriate approach to investigating the genome-wide transcription profile of HERV-H elements while at the same time taking each element into consideration individually. Although findings of this study did not represent the overall actual genome-wide expressional profile of HERV-H elements, we took the advantage of the PCR enrichment strategy to hook the most active HERV-H elements that produced transcripts containing the PBS-tRNA^His^ and 3′ R regions, and successfully identified the exact proviral loci of these elements in colon cancer cell lines and tissue samples. Expression patterns of HERV-H spliced transcripts from HERV-H elements were different among colon tumor, adjacent normal samples, and colon cancer cell lines.

Expression patterns of HERV-H spliced transcripts from HERV-H elements were different among the five colon cancer cell lines tested, evidenced by both gel electrophoresis of the RT-PCR products (Fig. 1B left) and sequencing of the amplicons after cloning (Table 1). Sequence analysis by comparing them with the constructed HERV-H consensus indicated that the active HERV-H elements found in this study were all structurally incomplete to some extent. This can also be inferred from the lengths, all of which were much shorter than the full-length HERV-H consensus (9.0-kb). The band patterns were different between SW480 and SW620 cells. Although they originated from the primary and metastatic tumors of the same patient, this difference between SW480 and SW620 may be just due to long-term culture. Likewise, the limited number of active HERV-H elements in each cell line may also be due to long-term culture in defined *in vitro* conditions. It is also interesting that by demethylation treatment using DNA methylation and histone deacetylase inhibitors, expression patterns in RKO and SW480 cells were changed significantly, suggesting that transcription of some HERV-H elements are regulated epigenetically.

Although no obvious difference was observed in the RT-PCR product bands between tumor and adjacent normal samples, the total numbers and loci of active HERV-H elements were significantly different (Fig. 3; Table 2). Seven HERV-H elements were found to be transcriptionally active in the adjacent normal colon samples. By comparison, 14 elements were found to be active in the tested colon tumor tissues. Expression of the three commonly most active elements (>10%) seemed to be ordinary; the one located at 1q24.2 produced 17.78% and 26.19% of the products in tumor and normal samples, and products from the one located at 16q24.1 consisted of 26.67% in tumor and 16.67% in normal samples, respectively. Interestingly, the one element located at 19q13.31 was the most active in adjacent normal tissues, producing 42.86% of the products, while only 11.11% from tumor samples belonged to 19q13.31. These results demonstrated that most of the HERV-H spliced transcripts in adjacent normal colon tissues were from 19q13.31, while transcripts in colon tumor tissues involved more elements.

All active HERV-H elements found in this study are structurally incomplete, with six fragments commonly deleted, which are distributed through the *gag*, *pol* and *env* regions (Fig. 2A); even so, some of them (40%) retain putative open reading frames (ORFs) (Tables 1 and 2; Fig. S1 and S2). Although some of these putative ORFs have been subjected to sequence deletion to a certain extent when compared with the corresponding region in the HERV-H consensus, seven of the putative peptide sequences (encoded by 6 HERV-H elements) contain conserved domains, with one conserved Protease domain, one conserved CREB5 (cAMP response element-binding protein 5) domain and five conserved reverse transcriptase (RT)-like domains. Interestingly, all the three commonly most active HERV-H elements (located at 1q42.2, 16q24.1 and 19q13.31) in tumor and adjacent normal tissues contain putative ORFs with conserved domains (Table 2; Fig. S2). Whether these ORFs actually produce proteins in colon tissues and what role they play in colon carcinogenesis are issues to be addressed.

The reason for the transcriptional difference in HERV-H elements between colon tumor and adjacent normal tissues remains unknown. Cells in tumor and adjacent normal samples are different in many aspects. Colon tumor cells in a sample are heterogeneous and cover more phases of the cell cycle, while non-tumor cells are usually in G0. However, there has been no study to address whether HERVs are expressed in a cell cycle dependent manner yet. Furthermore, tumor cells are in a more undifferentiated state as compared with non-tumor cells, reminiscent of the state of embryonic cells. Expression of HERVs in a tissue-specific and developmental stage-dependent manner has been reported [18]. HERV elements have also been found to be highly expressed in reproductive tissues such as testis and placenta, which contain undifferentiated cells [5], [19]. Whether HERV-H elements are expressed in a differentiation dependent manner, subsequently causing the difference between colon tumor and adjacent normal tissues, also needs to be investigated. These two points may indicate directions for further study on the mechanism by which HERV-H elements are differentially regulated between colon tumor and adjacent normal tissues.

This is the first attempt to study the characteristics of expression of HERV-H spliced transcripts in colon cancer. In summary, our results indicated that expression patterns of HERV-H elements differed among colon cancer cell lines and could be affected by demethylation treatment. More importantly, our findings demonstrated that HERV-H expression involved more active HERV-H elements in colon tumor than in adjacent normal tissues.

## Materials and Methods

### Ethics statement

This study was approved by the ethics committee of Zhejiang University, and written consents were obtained from all patients involved.

### Tissue samples, cell culture and RNA preparation

Colon tumor and adjacent normal tissue samples were obtained after surgical resection in the second affiliated hospital of Zhejiang University and stored frozen at −80°C until RNA extraction. Tissue types (tumor or normal) were assessed by histological staining. Cancer cells were obtained from American Type Culture Collection and grown in RPMI 1640 medium supplemented with 10% fetal calf serum. Total RNA was prepared with Trizol reagent (Invitrogen) according to the manufacturer's guide. RNA was always treated with RQ1 RNase-free DNase (Promega) before cDNA synthesis to eliminate genomic DNA contamination.

### Demethylation treatment with DNA methylation and histone deacetylase inhibitors

Cells were treated with the DNA methylation inhibitor 5-aza-2′-deoxycytidine (DAC; Sigma) and the histone deacetylase inhibitor trichostatin A (TSA; Beyotime) as described [20]: initial treatment with DAC (200 nM) for 48 h, with drug and medium replaced 24 h after the beginning of treatment, followed by the replacement of medium containing TSA (300 nM) for a further 24 h. Total RNA from drug treated cells or non-treated cells was isolated with Trizol reagent and treated with DNase before cDNA synthesis.

### Reverse transcription-PCR (RT-PCR)

RNA was reverse-transcribed into cDNA using M-MLV Reverse Transcriptase (Promega) according to the manufacturer's guide. PCR assays were performed with *Taq* DNA polymerase (Promega) in reaction systems containing 0.2 µmol/L forward and reverse primers each. Thermal cycler parameters were 94°C 5 min, (94°C 30 s, 55°C 30 s, 72°C 90 s)×36 cycles, 72°C 10 min. PCR products of each cell sample, PCR product mixture of six tumor samples and PCR product mixture of six adjacent normal samples were purified with AxyPrep PCR Cleanup kit (Axygen), cloned into the pGEM-T easy vector (Promega) and transfected into *E. coli* bacteria. Over 30 colonies from each cell sample and 100 colonies from tumor or normal tissue samples were subjected to Sanger sequencing. Targets of the primers are indicated in Fig. 1A and their nucleotide sequences are as follows: forward (targeting PBS-tRNA^His^): 5′-TGGTGCCGTGACTCGGAT-3′, reverse (targeting R region): 5′-GCTGAGTCCGAAAAGAGAGTC-3′. The primers were designed by referring to the HERV-H consensus constructed by Jern *et al.* [5], with “G” in the forward primer modified according to genome-wide sequence analysis on HERV-H-related primer binding sites on our own (not shown).

### Sequence analysis

Sequences were searched with BLAT method against human genome using the online server (http://genome.ucsc.edu/cgi-bin/hgBlat?command=start). Sequencing results were excluded if the sequences in-between the primers had more than five dissimilarities to genomic DNA. Genomic DNA sequences were retrieved from the human genome assembly (hg19) at http://genome.ucsc.edu/cgi-bin/hgGateway. Sequences were analyzed with the tool RepeatMasker at http://www.repeatmasker.org/cgi-bin/WEBRepeatMasker to identify repeat elements. Pairwise alignments were carried out with GeneDoc. ORF prediction was performed using the online program ORF Finder (http://www.ncbi.nlm.nih.gov/gorf/gorf.html). Then putative amino acid sequences were blasted against the non-redundant protein sequence database using the online BLASTP program (http://blast.ncbi.nlm.nih.gov/Blast.cgi).

### Sequence submission

A total of 92 nucleotide sequences of the newly identified HERV-H spliced transcripts have been deposited in dbEST with accession numbers of JK017330–JK017415 and JK475044–JK475049 (with 7 EST library accession numbers of LIBEST_027280 - 027286).

## Supporting Information

Figure S1 **Pair-wise alignments for each HERV-H element were performed with the HERV-H consensus constructed by Jern P,** ***et al*** **.** The shortened alignment results are shown to indicate the missing regions precisely. Color density represents the extent of homology with the HERV-H consensus. Lines represent deleted regions in the HERV-H elements as compared with the HERV-H consensus. Red rectangles indicate the regions where putative ORFs are harbored, while red rectangles with thick lines indicate ORFs with conserved domains. Regions of LTRs, pre-gag, *gag*, *pro*, *pol* and *env* are labeled below. Genomic locus of each HERV-H element is indicated correspondingly on the right side.
(TIF)

Figure S2 **Pair-wise alignments for each HERV-H element were performed with the HERV-H consensus constructed by Jern P,** ***et al*** **.** The shortened alignment results are shown to indicate the missing regions precisely. Color density represents the extent of homology with the HERV-H consensus. Lines represent deleted regions in the HERV-H elements as compared with the HERV-H consensus. Red rectangles indicate the regions where putative ORFs are harbored, while red rectangles with thick lines indicate ORFs with conserved domains. Regions of LTRs, pre-gag, *gag*, *pro*, *pol* and *env* are labeled below. Genomic locus of each HERV-H element is indicated correspondingly on the right side. The number of each element is shown on the left as indicated in Table 2.
(TIF)
