# Supplementary figures and images for: Expression Patterns of Non-Coding Spliced Transcripts from Human Endogenous Retrovirus HERV-H Elements in Colon Cancer
Source: PLoS One. 2012 Jan 6;7(1):e29950. doi: 10.1371/journal.pone.0029950 (PMC3253121; doi:10.1371/journal.pone.0029950)

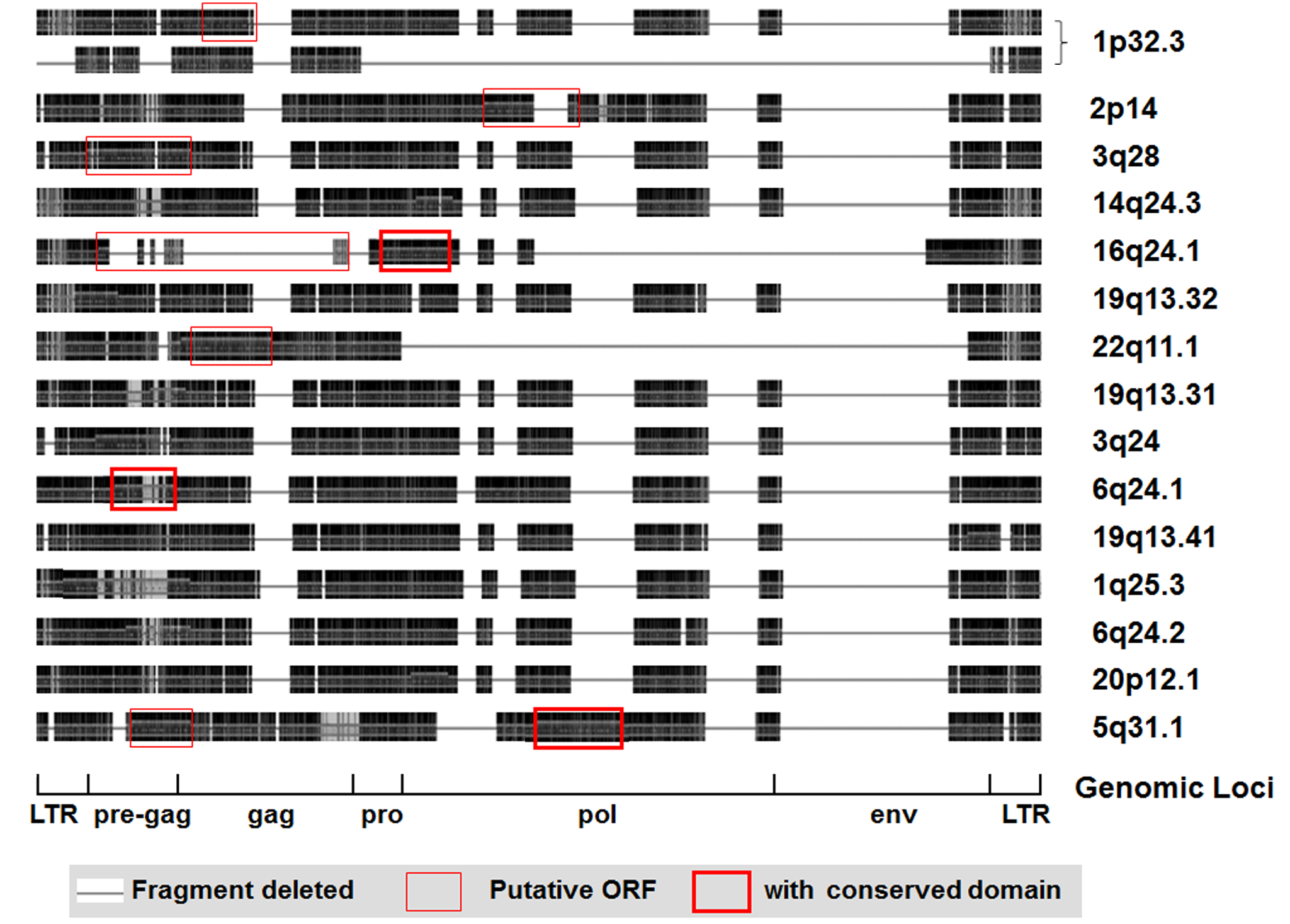

Supplement: Figure S1 — Pair-wise alignments for each HERV-H element were performed with the HERV-H consensus constructed by Jern P, et al . The shortened alignment results are shown to indicate the missing regions precisely. Color density represents the extent of homology with the HERV-H consensus. Lines represent deleted regions in the HERV-H elements as compared with the HERV-H consensus. Red rectangles indicate the regions where putative ORFs are harbored, while red rectangles with thick lines indicate ORFs with conserved domains. Regions of LTRs, pre-gag, gag, pro, pol and env are labeled below. Genomic locus of each HERV-H element is indicated correspondingly on the right side. (TIF) [file pone.0029950.s001.tif]

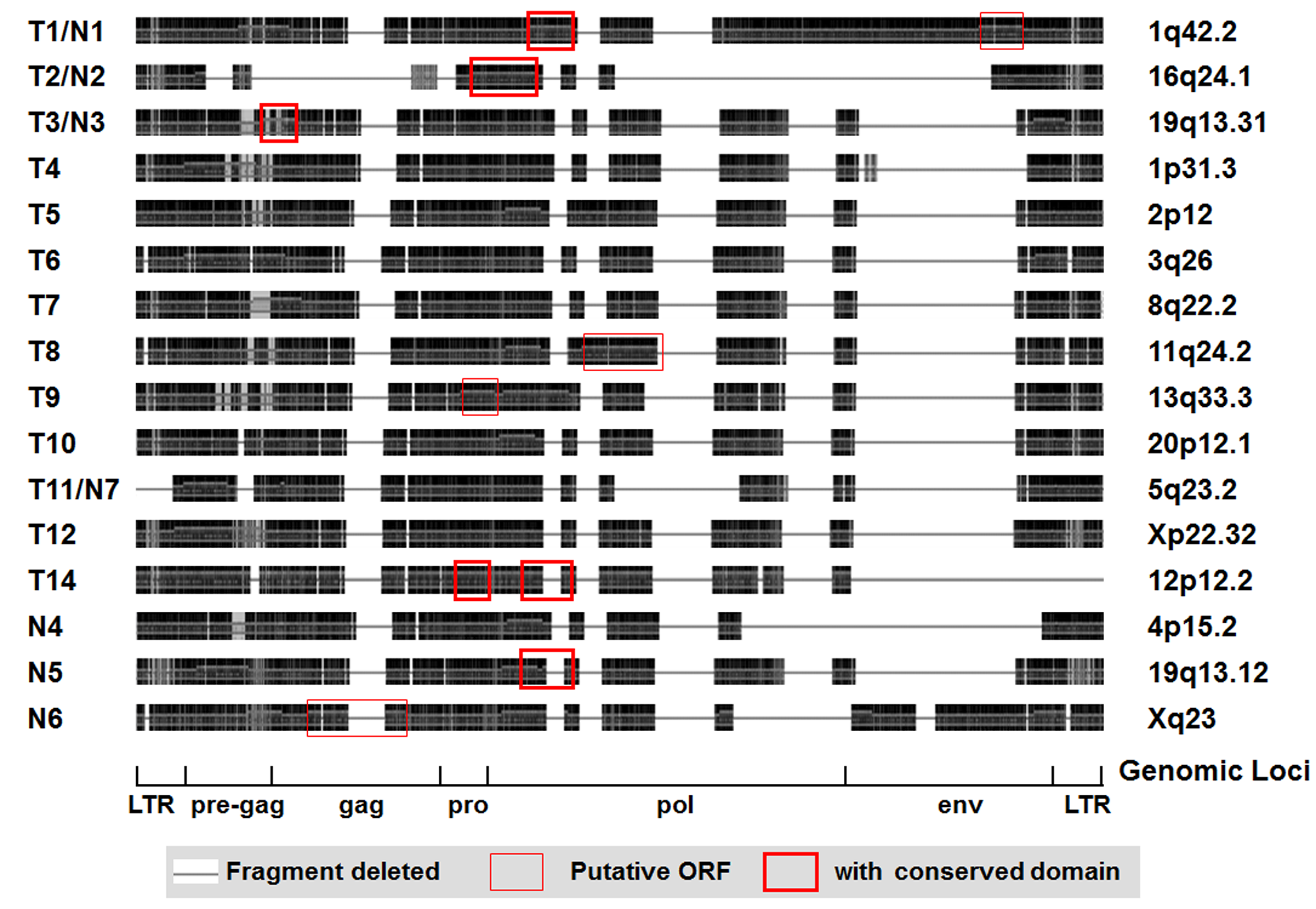

Supplement: Figure S2 — Pair-wise alignments for each HERV-H element were performed with the HERV-H consensus constructed by Jern P, et al . The shortened alignment results are shown to indicate the missing regions precisely. Color density represents the extent of homology with the HERV-H consensus. Lines represent deleted regions in the HERV-H elements as compared with the HERV-H consensus. Red rectangles indicate the regions where putative ORFs are harbored, while red rectangles with thick lines indicate ORFs with conserved domains. Regions of LTRs, pre-gag, gag, pro, pol and env are labeled below. Genomic locus of each HERV-H element is indicated correspondingly on the right side. The number of each element is shown on the left as indicated in Table 2. (TIF) [file pone.0029950.s002.tif]
